# Cyclizing-berberine A35 induces G2/M arrest and apoptosis by activating YAP phosphorylation (Ser127)

**DOI:** 10.1186/s13046-018-0759-6

**Published:** 2018-05-04

**Authors:** Wuli Zhao, Hong Liu, Junxia Wang, Mengyan Wang, Rongguang Shao

**Affiliations:** Key Laboratory of Antibiotic Bioengineering, Ministry of Health, Laboratory of Oncology, Institute of Medicinal Biotechnology, Peking Union Medical College and Chinese Academy of Medical Sciences, 1 Tiantan Xili, Beijing, 100050 China

**Keywords:** Dual topoisomerase inhibitor, Topoisomerase2α, G2/M arrest, DNA breakage, YAP1

## Abstract

**Background:**

A35 is a novel synthetic cyclizing-berberine recently patented as an antitumor compound. Based on its dual targeting topoisomerase (top) activity, A35 might overcome the resistance of single-target top inhibitors and has no cardiac toxicity for not targeting top2β. In this study we further explored the biological effects and mechanisms of A35.

**Methods:**

Antitumor activity of A35 was evaluated by SRB and colony formation assay. G2/M phase arrest (especially M) and first damage of M-phase cells were investigated by flow cytometry, cytogenetic analysis, immunofluorescence, co-immunoprecipitation and WB. The key role of phospho-YAP (Ser127) in decreasing YAP nuclear localization, subsequent G2/M arrest and proliferation inhibition were explored by YAP1^−/−^ cells, mutated Ser127 YAP construct (Ser127A) and TUNEL.

**Results:**

G2/M arrest induced by A35 was independent of p53. M phase cells at low dose were firstly damaged and most damaged-cells accumulated in M phase, and that was a result of preferring targeting top2α by A35, as top2α is essential to push M phase into next phase, and targeting top2α induced cells arrested at M phase. A35 decreased YAP1 nuclear localization by activating YAP phosphorylation (Ser127) which subsequently regulated the transcription of YAP target genes associated with growth and cycle regulation to induce G2/M arrest and growth inhibition.

**Conclusions:**

Our studies suggested the mechanism of G2/M arrest induced by A35 and a novel role of YAP1 (Ser127) in G2/M arrest. As a dual topoisomerase inhibitor characterized by no cardiac toxicity, A35 is a promising topoisomerase anticancer agent and worthy of further development in future.

## Background

Berberine (BBR), an isoquinoline natural product extracted from *Coptis chinensis*, has been extensively employed in anti-inflammatory [1], cholesterol-lowering [2] and antineoplastic [3] research, but its anticancer activity is weak [3, 4]. In search for cholesterol-lowering agents, we occasionally found the novel skeleton compound cyclizing-berberine (berberine of 1,13-cyclication), and further study showed that cyclizing-berberine and its derivatives have strong antitumor activities in liver, colon, lung, leukemia and breast cancer cells and cells resistant to doxycycline (DOX). Mechanistic studies showed that as a dual top (top1 and top2α), the inhibitor A35 preferentially and specifically targets top2α and has no effect on the cardiac toxicity inducer top2β. In vitro studies showed that A35 could intercalate into DNA but not interfere with DNA-top2α binding or top2α ATPase activity. it mainly disturbed the top2α catalytic cycle by intercalation between DNA-topoisomerase to enhance pre-strand and post-strand cleavage and inhibited DNA relegation to form the DNA-top2α cleavage complex. In vivo (cells and nude mice model) results revealed that A35 could facilitate DNA-top2α cleavage complex formation, double-stranded DNA breakage and apoptosis. However, the biological effects of A35 on cells have not been clarified entirely. In the present study, we focused on the effects of A35 on cell cycle distribution and its mechanism, DNA damage and apoptosis as well as the associated protein and signaling pathways underlying the above-mentioned biological effects.

In this study, we demonstrated that A35 could induce cells arrest at G2/M independent of p53, but further molecular mechanism still be obscure. Recent studies showed that YAP plays a key role in DNA damage, apoptosis and induction of cell cycle arrest, for example YAP could induce G0/G1 arrest by regulating the transcription of cell cycle-associated proteins [5–9]. But about the relationship between YAP and G2/M arrest, only one report demonstrated that YAP involved in the G2-M transition [10], and further studies about the role of YAP phosphorylation (Ser127) during G2/M arrest were not further reported.

In this study, we demonstrated that A35 could induce G2/M arrest, arrest mechanism and the arrested cells were DNA damage cells. A35 firstly induced DNA damage in M phase due to targeting top2α, and eventually validated DNA breakage by chromosome detection. The anticancer activity and G2/M arrest induced by A35 was independent of p53 and mainly dependent on the decreasing nuclear localization of YAP1 by activating YAP phosphorylation (Ser127), which subsequently reduces the transcription of YAP target genes associated with growth and cycle regulation.

## Methods

### Reagents and cells

Anti-γ-H2AX (Ser139), anti-phospho-ATM (Ser 1981), anti-phospho-DNA-PK(Ser2056), anti-phospho-BRCA1 (Ser 1524), anti-Cyr61, anti-survivin,anti-YAP, p-YAP (Ser127) and anti-CyclinB1 were purchased from Cell Signaling Technology (Danvers, MA, USA) or Abcam (Cambridge, MA, USA). Anti-γ-H2AX (Ser139), conjugated with fluorescein isothiocyanate (FITC) was purchased from BD Company (Franklin Lakes, New Jersey, USA). The anti-β-actin antibody, 7-Hydroxystaurosporine (UCN-01) and colcemid were purchased from Sigma-Aldrich (Saint Louis, Missouri, USA), and peroxidase-conjugated goat anti-mouse and goat anti-rabbit secondary antibodies were purchased from ZSGQ-BIOCompany (Beijing, China). 7-Hydroxystaurosporine (UCN-01) and colcemid were purchased from Sigma-Aldrich (Saint Louis, Missouri, USA).

### Cell lines

Human K562, HepG2, Raji, HCT116 and HCT116-KO cancer cells were obtained from either our lab or American Type Culture Collection (ATCC). K562 cells and Raji were cultured in 1640 medium supplemented with 10% fetal bovine serum (FBS), and HepG2, HCT116 and HCT116-KO were cultured in Dulbecco’s Modified Eagle’s Medium (DMEM) with 10% FBS at 5% CO_2_ and 37 °C. Cells in the exponential growth phase were harvested with a 0.25% trypsin-0.02% EDTA solution and resuspended in the specified medium. Only single cells with viabilities over 95% (trypan blue exclusion) were used. HCT116 cells were transfected by YAP CRIPSER plasmid (Santa Cruz Biotechnology, Santa Cruz, CA, USA) and YAP^−/−^ HCT116 cells were screened by puromycin and Western Blot.

### Cell growth inhibition assay

Cell growth inhibition was determined using a sulforhodamine B (SRB) assay as previously described [11]. Cells were seeded in 96-well plates at 4 × 10^3^-8 × 10^3^ cells/well, treated with increasing concentrations compound for indicated time, and then fixed with 50% trichloroacetic acid (Sigma-Aldrich,Saint Lois, Missouri, USA), and finally 0.4% (*w*/*v*) SRB in acetic acid (1%) was added. SRB bound cells were solubilized with 10 mM Trizma base. The absorbance was read at 492 nm. Growth inhibition (%) was calculated at each concentration, and the IC50 was calculated by SigmaPlot. Assays were repeated three times, and the results are presented as the mean ± SD.

### Analysis of cell cycle distribution

Flow cytometric analysis was performed as described in our previous report [11]. HCT116 cells were treated with 2 μM A35 for indicated time, and then harvested and fixed with 75% ethanol at − 20 °C overnight. Cells were stained with propidium iodide (100 μg/mL) and RNase A (200 μg/mL) at 37 °C for 30 min. The DNA content was analyzed with a FACScan flow cytometer (COULTEREPICS XL, Fullerton, Cal, USA).

### Quantification of the mitotic index

Because the phosphorylation of histone H3, one of four core histone proteins (H2A, H2B, H3 and H4) that packs DNA in nucleosomes, Phosphorylation of Histone H3 at Ser10 is tightly correlated with chromosome condensation during mitosis [12]. Hence, Histone H3 pSer10 signal indicates the mitotic cell. For this assay, treated cells were incubated with 0.5% Triton for 20 min and blocked with FBS at 37 °C for 30 min, and an anti-histone H3 (pSer10) antibody was then added overnight at 4 °C as previously described [13]. After adding a secondary antibody conjugated with FITC for 2 h, slides were mounted with 4′,6-diamidino-2-phenylindole (DAPI, Sigma-Aldrich (Saint Louis, Missouri, USA). Histone H3 (pSer10)-positive cells were counted at 400× magnification, and the total numbers of nuclei were determined for each section. At least 200 cells per field in a minimum of three randomly selected fields were counted on three slides for each sample.

### Western blot

Whole-cell lysates were used for immunoblotting as described previously [14]. Cells were lysed using lysis buffer (50 mmol/L Tris-HCl (pH 8.0), 150 mmol/L sodium chloride, 1.0% Triton X-100, 0.5% sodium deoxycholate, 0.1% SDS, protease inhibitor), and 20 μg of protein lysate was resolved by SDS-PAGE and analyzed by immunoblotting with specified antibodies. Immunoreactive signals were revealed using the enhanced chemiluminescence method (Millipore) and visualized with a ChemiImager 5500 imaging system (Alpha Innotech).

### Immunofluorescence

Treated cells were incubated with 0.5% Triton for 20 min, blocked with FBS at 37 °C for 30 min, and then mixed with a γ-H2AX antibody conjugated with FITC or another primary antibody overnight at 4 °C according to a previously established protocol [15]. A secondary antibody conjugated to FITC was then added for 2 h, and the slides were mounted with DAPI and observed with Laser scanning Confocal Microscopy. γ-H2AX-positive cells were counted at 400× magnification to obtain the total number of nuclei per section. Five microscope views from each sample were counted and averaged.

### Cytogenetic analysis

Colcemid (100 ng/mL) was added to cell cultures 2 h prior to harvest. Cells were collected and swollen in 0.075 mol/L KCl at 37 °C for 10 min and then fixed in fresh methanol/acetic acid (3:1, *v*/v) at room temperature for 20 min. After three washes with ice-cold methanol/acetic acid (3:1, v/v), cells were spread onto slides and air-dried overnight. Spreads were stained with DAPI at room temperature for 10 min, and at least 50 metaphase spreads were analyzed on three separate slides for each sample. For each treatment, variations between slides were not remarkable, as determined by statistical assays (data not shown).

### TUNEL

Drug-treated cells were used for the terminal deoxynucleotidyl transferase dUTP nick-end labeling (TUNEL) assay as previously described [16].

## Results

### A35 prohibits cancer cell proliferation in a dose- and time- dependent manner

**Fig. 1:**
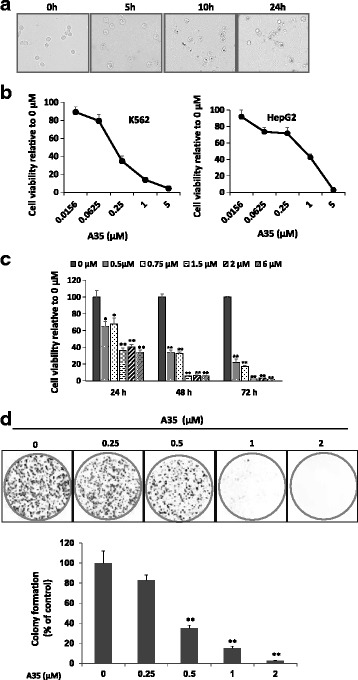
A35 suppresses cancer cell proliferation in a time- and dose-dependent manner. **a** The cell morphology of human leukemic K562 cells was observed at 5, 10 and 24 h after treatment with 1 μM A35. **b** K562 and HepG2 cells were treated with the indicated concentrations of A35, and cell survival was detected by the SRB assay. A dose-dependent curve was depicted. **c** Vitality of K562 cells after treatment with 0.5, 0.75, 1.5, 2 and 6 μM A35 for 24, 48 and 72 h. A time-dependent curve was plotted. **d** HepG2 cells were seeded in a 6-well plate at 3000 cells/well. After 24 h, various concentrations of A35 were added, and the cells continued to incubate for 7 days. Visible colonies were then counted as described in the materials and methods. Colony formation rate = (number of colonies/number of seeded cells) × 100%. *P < 0.05; ** *P* < 0.01

To evaluate the effects of A35 on tumor cells, 1 μM A35 were added into the human leukima cells K562 and cell morphology was observed at 5, 10 and 24 h. The cells were enlarged at 5 h, and dark dots were observable for up to 10 h, especially in the nucleus, perhaps due to the aggregation of A35 in the cell nucleus (the target of A35 was topoisomerase localized in the nucleus) (Fig. 1a). When the cells were treated for up to 24 h, some broken cells and cell debris were observed. After 48 h of treatment, most K562 cells had disappeared, and large amounts of cell debris were visible (data not shown).

Cell vitality was detected after incubation with increasing doses of A35 for 48 h, showing that A35 significantly inhibited cell growth in a dose-dependent manner. For K562 cells treated with A35 at 0.0625 μM, cell survival was approximately 80% of that of the control after 48-h treatment. This number decreased to 35% with 0.25 μM A35, and cell vitality with 5 μM A35 was approximately 5% (Fig. 1b, left). Similarly, the anticancer effects of A35 were also observed in HepG2 cells in a concentration-dependent manner (Fig. 1b, right). Meanwhile, we examined the survival of K562 cells after treatment with A35 for varying time points (24, 48 and 72 h), and obvious time-dependent results were observed (Fig. 1c).

Colony formation assay (CFA) is an in vitro cell survival assay based on the ability of single cells to grow into colonies, and that is the gold standard of cytotoxic agent effects evaluation and more stringent tests for malignant proliferation [15, 17, 18]. Thus we introduced colony formation assay to further assess the anticancer effect of A35, and by this assay we could more stringently clarify antitumor characterization of A35. HepG2 cells were seeded in a 6-well plate at 3000 cells/well and incubated with increasing concentrations of the compound for 7 days prior to colony counting. A35 could inhibit the colony formation of HepG2 cells in a dose-dependent manner (Fig. 1d), indicating its high ability to reduce cancer growth.

### A35 leads to significant G2/M phase arrest, especially M phase arrest, independent of p53 in various cancer cells

**Fig. 2:**
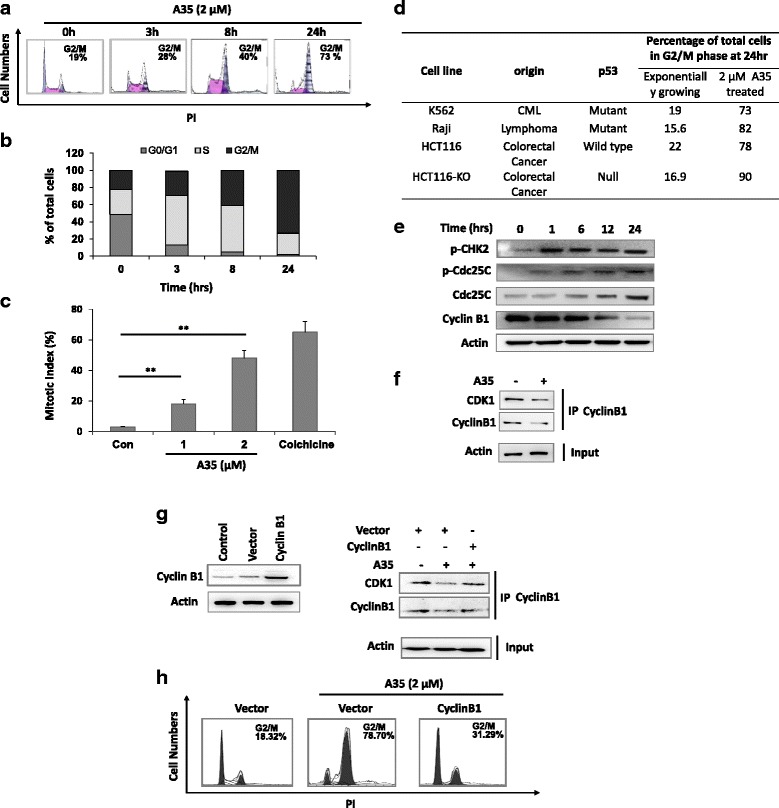
A35 leads to significant G2/M phase arrest in different cancer cells independent of p53. **a** DNA content in K562 cells treated with 1 μM A35 for 0, 3, 8 and 24 h as determined by propidium iodide staining and flow cytometry. **b** Quantitative analysis of (**a**). **c** K562 cells were incubated with colchicine or A35 (0.5 or 1 μM) for 24 h. Cells were subjected to immunofluorescence with histone H3 (pSer10), and the mitotic index was calculated. **d** Cancer cell lines of diverse origins expressing wild type p53, mutated p53 or deleted p53 were treated with 1 μM A35 for 24 h. DNA content was analyzed by propidium iodide staining and flow cytometry. **e** HCT116 cells were treated by A35 for the indicated time and G2/M arrest-associated proteins were detected by immunoblot. **f** HCT116 cells were treated for 24 h with A35 and CyclinB1/CDK1 complex was detected by co-immunoprecipitation assay. Cyclin B1 construct was transfected into HCT116 cells, after 24 h A35 was added to incubate for an additional 24 h, and CyclinB1/CDK1 complex was detected by co-immunoprecipitation (**g**), and cell cycle was detected by flow cytometry (**h**).*P < 0.05; ** *P* < 0.01

The effect of A35 on cell cycle distribution was determined by DNA content analysis in K562 cells at the indicated time points after A35 treatment (Fig. 2a). The ratio of G2/M phase cells was slightly elevated at 3 h, and the proportion of cells in the G2/M phase increased with treatment time, eventually up to 73% of all cells at 24 h. These data indicated that A35 could induce G2/M phase arrest in a time-dependent manner. To distinguish G2 cells from M cells (mitotic cells), we conducted microscopic evaluation to detect the ratio of histone H3 (pSer10)-positive (marker of mitotic cells) cells by immunofluorescence, obtaining the mitotic index. The numbers of cells in M phase were increased significantly after treatment with 1 μM and 2 μM A35, and at 2 μM the ratio of M-phase cells to total cells was about 48% (Fig. 2c) and the G2/M cells ratio was 73% to total cells (Fig. 2a). These data indicated that approximately 70% G2/M arrest cells were M-phase cells; thus, A35 predominantly caused M phase arrest.

Given that p53 plays a key role in G2 and M arrest in response to DNA damage [19, 20], we investigated whether the G2/M phase arrest induced by A35 was dependent on p53. Cells expressing wild-type p53, mutated p53 and deleted p53 were employed to assess G2/M arrest, revealing that all type of cells remained blocked at the G2/M phase regardless of p53 status (Fig. 2d). These results indicated that the G2/M arrest induced by A35 was independent of p53.

To elucidate the molecular basis of A35-induced G2/M arrest, we first investigated activation of the Chk2-Cdc25C-CyclinB G2/M checkpoint pathway. Upon incubation of HepG2 and K562 cells with 2 μM A35, the DNA damage checkpoint kinase Chk2 became phosphorylated within 1 h, indicating its activation (Fig. 2e). This was associated with increased phosphorylation of the Chk2 target protein Cdc25C on Ser216 and decreased CyclinB1. In addition, p53 and p21 levels are not obviously elevated after 24 h of A35 treatment based on a previous study [16]. Thus, activation of p53 may not be required for A35-induced G2/M arrest.

As we known the complex CyclinB1/CDK1 formation is the essential player in G2/M transition, and the decrease of CyclinB1/CDK1 formation will induce G2/M arrest. Our results showed that the CyclinB1 expression (one factor of the complex) reduced in the presence of A35 (Fig. 2e), and that also hinted the formation of CyclinB1/CDK1 complex might be reduced, and this decrease will prevent G2-M transition and then G2/M arrest was induced. To validate CyclinB1/CDK1 complex formation indeed lessened in the presence of A35, we performed co-immunoprecipitation assay with CyclinB1 antibody to detect whether CDK1 was pulled down. Results showed that after A35 treatment CDK1 protein to be pulled down lessened (Fig. 2f), indicating the CyclinB1/CDK1 formation reduced indeed.

To further elucidate the decrease of CyclinB1-CDK1 complex formation play the key role in G2/M arrest induced by A35, we forced CyclinB1 high expression and detect whether G2/M arrest was reverted when CyclinB1-CDK1 complex formation increased, and results turned out the G2/M arrest induced by A35 was reversed (Fig. 2g-h), indicating that CyclinB1-CDK1 complex play crucial role in G2/M arrest induced by A35.

### A35 firstly leads to DNA breakage of M phase cells due to mitosis inhibition induced by A35-targeting top2α

Given that many DNA-damaging agents induce G2/M phase arrest to repair broken DNA [21, 22], and A35 is a topoisomerase inhibitor and can induce DNA breakage, we wanted to verify whether DNA damage cells were G2/M-arrested cell.

**Fig. 3:**
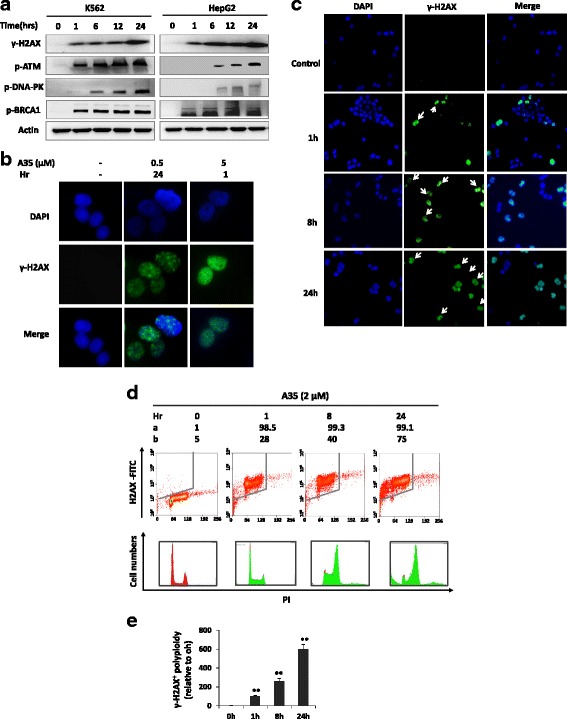
A35 induces DNA breakage in M-phase cells firstly due to mitosis inhibition by targeting top2α. **a** K562 cells and HepG2 cells were incubated with 2 μM A35, and samples were collected at the indicated time points to detect DNA damage-associated proteins by immunoblot. **b** HCT116 cells were treated with the indicated concentrations of A35 for 1 h or 24 h, and cells were fixed and stained by a FITC-labeled Ser139-phosphorylated H2AX (γ-H2AX) antibody. Representative images were acquired by a confocal microscope. **c** K562 cells were treated with 0.5 μM A35, and γ-H2AX-positive cells were detected at various time points by a confocal microscope. **d** K562 cells were treated with 2 μM A35, and γ-H2AX-positive cells were detected by flow cytometry at the indicated time points. γ-H2AX-positive polyploidy cells were counted (**e**). **a**: Percentage of γ-H2AX-positive cells among total cells. **b**: Percentage of γ-H2AX-positive G2 cells among total γ-H2AX-positive cells. *P < 0.05; ** *P* < 0.01

As γ-H2AX is an important marker of double-stranded breaks [23, 24], we detected its level, finding that H2AX was rapidly phosphorylated at Ser139 (γ-H2AX) in response to DNA double-stranded breaks. Immunoblotting showed that γ-H2AX expression in K562 and HepG2 cells was induced after 1 h treatment with A35, and its increase was time-dependent (Fig. 3a). Then, we utilized confocal microscopy to examine whether γ-H2AX foci were formed (γ-H2AX foci represent DNA damage sites in the nucleus) in the presence of A35 [25], and results revealed that A35 could cause the formation of γ-H2AX foci in the nuclei of HCT116 cells after 1 h at a higher dose and after 24 h at a lower dose. These data indicated that A35 could not only induce DNA breakage in a short amount of time but also that the damage was persistent in response to A35 (Fig. 3b).

DNA damage usually evokes repair-associated pathways, and activation of these pathways is attributed to the repair of broken DNA. We examined repair-associated proteins, revealing that the DNA damage sensors ATM, DNA-PK and BRCA1 were strongly activated after incubation with A35 for 1 h. These activations were persistent over 24 h and were time-dependent in response to A35 to promote the repair of broken DNA.

Next, we wanted to identify cells in which phase of cell cycle were firstly damaged, and a lower dose of A35 (0.5 μM) was administered to K562 cells to avoid nonspecific targeting. Results showed that mitotic cells were damaged firstly compared with cells in other phases of cell cycle (Fig. 3c), and the numbers of γ-H2AX-positive G2/M cells increased in a time- and dose-dependent manner (Fig. 3c). Given that topoisomerases play essential roles in DNA replication and transcription in any phase of cell cycle, A35 could damage cells in all phases of the cycle by targeting topoisomerase. However, as top2α and top1 share common actions in the relaxation of single DNA strands, they could compensate for each other’s functions in G1-, S- and G2-phase cells. As A35 dominantly targeted top2α and lightly inhibited top1, we speculated that although top2α activity was blocked by A35, top1 could substitute top2α function to relax DNA and execute subsequent transcription or replication in G1-, S- and G2-phase. However, in M-phase cells, only top2α can drive the separation of two DNA duplexes after replication and top1 could not substituted top2α function, so top2α are lethal for M-phase progression and cell survival [26, 27]. Thus, we inferred that A35 firstly damaged M-phase cells due to the non-substitutable activity of top2α.

Next, a higher dose of A35 (2 μM) was added to cells, and flow cytometry showed that the almost all DNA of cells was rapidly damaged after 1 h treatment, and most of the damaged cells accumulated in M phase. Up to 24 h, some of the damaged cells underwent apoptosis (Fig. 3d). Similarly, polyploidy cells characterized by γ-H2AX positivity were observed, and the numbers of polyploidy cells increased in a time-dependent manner (Fig. 3d-e). These results indicated that DNA damage cells were dominantly blocked at M phase, and cell division was prohibited after treatment with A35.

### A35 induces chromosomal aberration

**Fig. 4:**
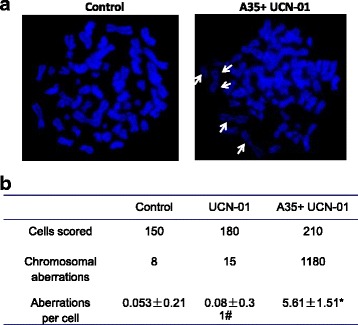
Chromosome aberrations induced by A35. K562 cells were treated with 2 μM A35 for 24 h, 100 nM UCN-01 was added, and culturing continued for 6 h to abrogate M-phase arrest prior to sample collection. Colcemid (100 ng/mL) was then added to the cell cultures 2 h prior to harvesting. **a** Representative images of chromosome spreads. Arrows, chromosome breaks. **b** Quantitation and comparison of chromosome aberrations. *p* < 0.01 vs Control or UNC-01 alone, # *p* = 0.1 vs Control

Based on the abovementioned results, the formation of double-stranded breaks was the main factor underlying the anticancer activity of A35. To visualize DNA damage more intuitively, consistent with a previous report [13], we conducted cytogenetic analysis of K562 cells treated with colcemid to force cells to remain in metaphase. This allowed the observation of chromosomal damage, as chromosomes are relaxed and damage is easily observed only in metaphase [28–30]. UCN-01, a Chk1 kinase inhibitor, was used to abrogate A35-induced G2 arrest [13]. This inhibitor allows G2-arrested cells to proceed through mitosis and into metaphase for the observation of chromosome damage, and treatment with 100 nM UCN-01 alone had no discernible effects on cell cycle distribution (Fig. 4a). By scoring 50 well-resolved metaphases, we detected an average of five chromatid type aberrations, including gaps and breaks, per cell (Fig. 4b). In contrast, fewer aberrations were found in control cells and cells treated with UCN-01 alone (data not shown).

### Proliferation inhibition and apoptosis induced by A35 are dependent on YAP phosphorylation (Ser127)

Activation of p53 and its downstream pathway plays an important role in apoptosis in response to DNA damage in most DNA-damaging agents. Our previous study revealed that the topoisomerase-targeting compound A35 did not activate p53, but the serious DNA damage response and sequent apoptosis were observed [16]. Thus, we inferred that there were other proteins or pathways to participate in DNA damage response and apoptosis after A35 treatment.

The Hippo pathway plays an important role in cell proliferation, differentiation and DNA damage, and YAP is the major downstream effector of the Hippo pathway [31–33]. Some studies showed that YAP participated in DNA damage-induced apoptosis and is the key role in tumor suppression independent of p53, YAP knockdown sensitized tumor cells to chemotherapy and radiation effects via increased accumulation of DNA damage [14, 34].

High YAP activity plays a critical role in the tumorigenesis of many human cancers, and YAP knockdown inhibits tumor proliferation [35]. Regulation of YAP activity depends on its nuclear transportation, and nuclear localization of YAP promotes transcription of its target genes associated with growth [33]. Ser127 phosphorylation is the critical factor responsible for the translocation of YAP between the nucleus and cytoplasm, and phosphorylated YAP (Ser127) binds partner cytoplasmic proteins and stays in the cytoplasm. When Ser127 phosphorylation level decreases, YAP can’t bind its partner proteins and translocate to the nucleus, triggering transcription of target genes and promoting proliferation and corresponding biological responses [31].

**Fig. 5:**
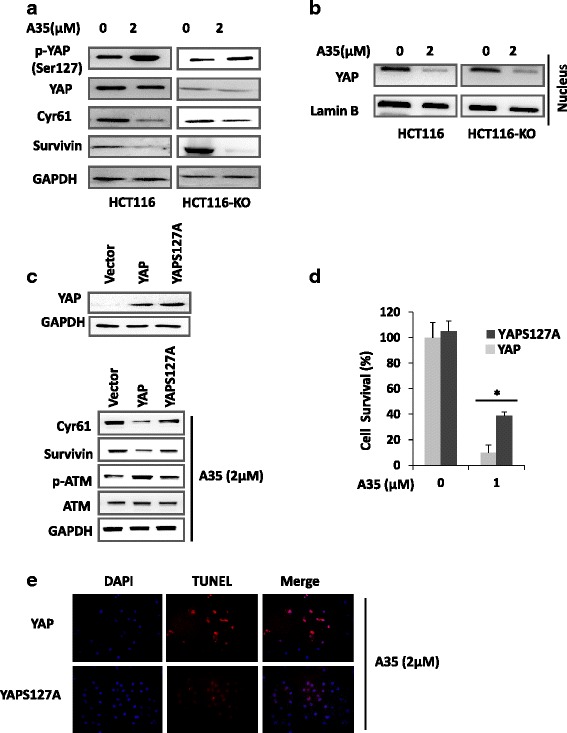
Proliferation inhibition and apoptosis induced by A35 are dependent on YAP phosphorylation (Ser127). HCT116 and HCT116-KO cells were treated with A35 for 24 h, and whole proteins were detected by immunoblot with indicated antibodies (**a**). Nuclear and cytoplasmic proteins were extracted and detected by immunoblot (**b**). YAP and YAPS127A constructs were transfected into YAP-HCT116^−/−^ cells, and after 24 h A35 was added for an additional 24 h to detect the indicated proteins (**c**) and for 48 h to detect cell survival by the SRB assay (**d**) and apoptosis by TUNEL assay (**e**). *P < 0.05; ** *P* < 0.01

In this study, we used HCT116 (p53 wt) and HCT116-KO (p53 deletion) cells to detect YAP-associated pathway after an addition of A35. And results showed that the total levels of YAP and its target genes (Cyr61 and Survivin) all decreased, but its phosphorylation (Ser127) level increased (Fig. 5a).

Given that higher YAP phosphorylation (Ser127) might hinder its nuclear localization and subsequently inhibit target gene expression to inhibit proliferation and trigger apoptosis, we examined YAP nuclear localization. The nuclear YAP levels were obviously reduced in both HCT116 and HCT116-KO cells, and the decreased degree of nuclear YAP was higher than that of total YAP (Fig. 5b). To further investigate the effect of Ser127 phosphorylation on DNA damage, cell growth and apoptosis induced by A35, either a mutated Ser127 phosphorylation YAP construct (S127A, YAP Ser127 mutated to alanine) or a wild-type YAP construct was transfected into YAP^−/−^ HCT116 cells (YAP was knocked out) for 24 h, and A35 was added for an additional 24 h. The proliferation inhibition and apoptosis induced by A35 were reverted in YAP (S127A)-transfected cells compared with wild type YAP-transfected cells (Fig. 5d-e), correspondingly the decrease of YAP target proteins associated with growth and the increase of p-ATM were also reversed (Fig. 5c).

### G2/M arrest induced by A35 is dependent on YAP phosphorylation (Ser127)

Generally, in response to DNA damage p53 is activated and its stabilization will enhance, then p53 would transport into nuclear to regulate the transcription of cell cycle-associated protein to induce G2/M arrest and apoptosis, such as p53 could promote Cdc25C and p21 expression by transcription regulation, and then p21 induce cyclinB1 expression decrease to induce G2/M arrest [36–38].

In our assays we did not observe the increase in p53 and p21(p53 target protein) expression, but visualized the expression changes of p53 and p21 target protein, such as the expression increase of Cdc25C and the decrease of CyclinB1, and that were crucial to the inducer of G2/M block. Thus we speculated there were other proteins to regulate the transcription of G2/M arrest-associated protein to induce G2/M block.

Just mentioned-above that YAP involved in lots of DNA damage events and was the crucial role in apoptosis induction, and as an essential event among DNA damage response, some studies also reported that YAP participated in cell-cycle arrest, such as G0/G1 arrest by regulating the transcription of cell cycle-associated proteins, [5–9]. But about the role of YAP in G2/M arrest, only one report demonstrated YAP involved in G2/M transition [10], and the studies about the role of YAP phosphorylation (Ser127) during G2/M arrest were not reported.

Nuclear YAP is crucial to the regulation of cell cycle-associated protein transcription, and YAP phosphorylation (Ser127) play the important role in shift between nucleus and cytoplasm. Our above results indicated that the nuclear YAP lessened and CyclinB1 decreased (a key role to induce G2/M arrest) independent of p53 in the presence of A35, thus we inferred that the transcription changes of G2/M arrest-associated protein to induce G2/M arrest might be related with the nuclear YAP decrease.

**Fig. 6:**
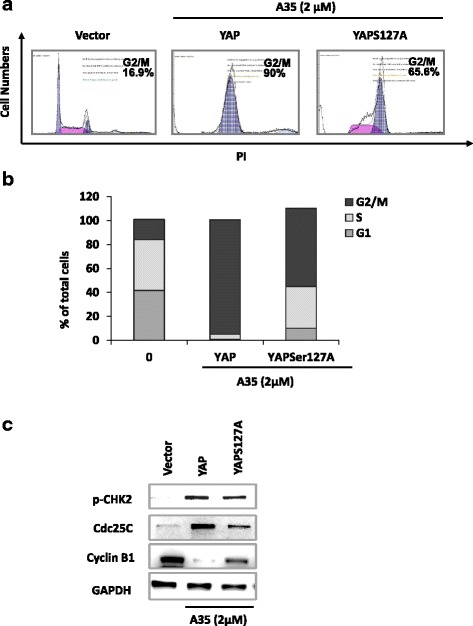
G2/M arrest induced by A35 is dependent on YAP activation. YAP and YAPS127A constructs were transfected into YAP-HCT116^−/−^ cells, and after 24 h A35 was added for an additional 24 h to analyze the DNA content by flow cytometry (**a**), and quantitative analysis of (**a**) (**b**), and cell cycle-associated proteins analysis by immunoblot (**c**)

Then we assessed the effects of YAP Ser127 phosphorylation on the G2/M arrest induced by A35, and results revealed that G2/M arrest in YAP (S127A)-transfected cells was abolished compared with wild-type YAP -transfected cells (decreased from 90 to 60%, Fig. 6a-b). Meanwhile, the signaling pathway promoting G2/M arrest including activation of Chk2 and the decrease of CyclinB1 in YAP mutated (S127A) cells were also alleviated compared with wild-type YAP.

## Discussion

Our previous study showed that A35 could target top1 and top2α to induce DNA-topoisomerase complex formation, mediating DNA breakage both in vivo and in vitro [16], but in vivo biological responses and associated pathways were not elucidated clearly. In this study, we further ascertained the anticancer activity of A35 by in a dose- and time-dependent manner on hematological and solid malignancies, and found A35 could induce G2/M arrest in a time-dependent manner and DNA of these arrested cells were damaged, and molecular pharmacological studies indicated the Chk2-Cdc25C-CyclinB1 pathway was provoked and CDK1/Cyclin B1 complex formation decreased to induce G2/M arrest. Meanwhile, the arrest was independent of p53 status, even though p53 has been shown to play a key role in induction of G2/M arrest in response to DNA damage. Correspondingly, p53 was not phosphorylated on Ser15, and increased p21 expression was not observed in A35-treated cells, as described in our previous study [16]. These data verified that p53 and p21 are not required for the onset of G2/M checkpoint activation and indicated the existence of a different mechanism involved in G2/M arrest.

Given that the dominant mechanism of A35 to inhibit cell growth is to target top2α to induce DNA breakage, we speculated that G2/M arrest and the corresponding pathway activation were a response to DNA breakage, and the detection of γ-H2AX foci (marker of DNA double-stranded breaks) also verified DNA damage induced by A35. In addition, DNA damage sensors, such as ataxia-telangiectasia mutated (ATM) and ataxia-telangiectasia mutated and Rad3-related (ATR), were well activated in response to DNA double-stranded breaks, further verifying DNA damage. Although in the previous paper [16] we also examined the DNA damage with immunofluorescence and comet assays, in the present study we further utilized laser scanning confocal microscopy to detect γ-H2AX foci formation since γ-H2AX foci represents DNA damage sites in the nucleus and its detection could quality the DNA damaged degree in a cell [13, 39], meanwhile we used flow cytometry to quality DNA breakage of all cells, and these approaches are more accurate for damage determination compared with immunofluorescence and comet assays mentioned in our previous manuscript.

Further analysis of cells arrested in the G2/M phase suggested that most of the arrested cells were in M phase. As mentioned above, both top1 and top2α function in the relaxation of single DNA strands, and they could compensate for each other’s functions in G1-, S- and G2-phase cells. At low dose, given that A35 dominantly targeted top2α and lightly inhibited top1, A35 might have an imperceptible DNA-damaging effect on G1-, S- and G2-phase cells thanks to compensatory effect of top1. When DNA replication was completed and cells proceeded to M phase, only top2α could separate and concentrate sister chromatids by relaxing and supercoiling double-strand DNA, targeting top2α by A35 would destroyed the relegation of relaxed or supercoiled DNA to inhibit chromatid concentration and separation, thus DNA damage might firstly observed in M-phase cells. Also we observed after A35 treatment more and more cells were blocked in M phase, and the arrested cells were unable to advance to the next phase of the cycle duo to the inhibition of chromatid concentration and separation. When a high dose of A35 was added, top1 was also targeted, and DNA of all phase cells was damaged thanks to activity inhibition of top1 and top2α. Furthermore, as A35 is a DNA intercalator, a large portion of A35 might intercalate into the broken ends of DNA during DNA relaxation mediated by top1 or top2α and induce non-specific DNA damage. Although the DNA-damaging repair pathway were activated to repair broken-DNA to proceed into the next phase of cell cycle, due to the inhibition of chromatid separation and concentration by targeting top2α, and broken cells would be arrested in G2/M and did not pass to a new G1 phase.

Our results showed that p53 was not involved in G2/M arrest or apoptosis, even though it has been shown to be an essential inducer of G2/M arrest and apoptosis in response to DNA damage [40]. A recent study showed that YAP1 is amplified in a vast array of solid tumors, including brain, colon and hepatocellular carcinomas, and it has been consistently reported as an oncogene in epithelial cancers [41]. High YAP activity plays a critical role in the tumorigenesis of many human cancers, and YAP knockdown inhibits tumor proliferation. Some reports showed that YAP participates in DNA damage response and is a key regulator of apoptosis independent of p53 [33]. Our results also indicated that the expression of YAP and its target gene decreased after A35 treatment. Further detection of nuclear extracts showed that the level of nuclear YAP and its target gene was drastically reduced to inhibit tumor proliferation.

Ser127 phosphorylation of YAP is the critical factor underlying the translocation of YAP between the nucleus and cytoplasm. Phosphorylated YAP (Ser127) binds cytoplasmic partner proteins and then retained in cytoplasm. However, when Ser127 phosphorylation of YAP decreases, YAP is released by its cytoplasmic partner protein and translocates into the nucleus, regulating the transcription of the target gene associated with growth and cell growth [31]. Mutation of Ser127 on YAP reverted proliferation inhibition, apoptosis and G2/M arrest induced by A35 compared with wild-type YAP. The underlying molecular mechanism in YAP (S127A) cells also supported above results and indicated that phosphorylated YAP (Ser127) could regulate proliferation-associated pathway and G2/M arrest-associated protein to play its anticancer activity, and phosphorylated YAP (Ser127) is the essential role in anticancer action of A35.

In summary, the novel skeleton compound cyclizing-berberine A35, which has an inhibitory mechanism on the top2α catalytic cycle different from that of other topoisomerase inhibitors [16], could strongly inhibit cancer proliferation and induce G2/M arrest, especially M-phase arrest. The ability of A35 to induce G2/M arrest and apoptosis were independent of p53 and instead dependent on the activation of YAP phosphorylation (Ser127). The subsequent repression of YAP nuclear translocation depressed cancer-promoting gene transcription and activation of a G2/M arrest-associated pathway. As a dual topoisomerase inhibitor characterized by no cardiac toxicity, A35 is a promising topoisomerase anticancer agent and worthy of further development in the future.

## Abbreviations

ATM: Ataxia-Telangiectasia
ATR: Ataxia Telangiectasia and Rad3 related
BBR: Berberine
DOX: Doxycycline
Top: Topoisomerase
UCN-01: 7-Hydroxystaurosporine

## References

[1] Kuo CL, Chi CW, Liu TY (2004). The anti-inflammatory potential of berberine in vitro and in vivo. Cancer Lett.

[2] Hwang JM, Wang CJ, Chou FP, Tseng TH, Hsieh YS, Lin WL, Chu CY (2002). Inhibitory effect of berberine on tert-butyl hydroperoxide-induced oxidative damage in rat liver. Arch Toxicol.

[3] Letasiova S, Jantova S, Cipak L, Muckova M (2006). Berberine-antiproliferative activity in vitro and induction of apoptosis/necrosis of the U937 and B16 cells. Cancer Lett.

[4] Lin JP, Yang JS, Lee JH, Hsieh WT, Chung JG (2006). Berberine induces cell cycle arrest and apoptosis in human gastric carcinoma SNU-5 cell line. World J Gastroenterol.

[5] Chen M, Wang J, Yao SF, Zhao Y, Liu L, Li LW, Xu T, Gan LG, Xiao CL, Shan ZL (2017). Effect of YAP inhibition on human leukemia HL-60 cells. Int J Med Sci.

[6] Wen Y, Ji Y, Zhang Y, Jiang B, Tang C, Wang Q, Chen X, Jia L, Gu W, Xu X (2017). Knockdown of yes-associated protein induces the apoptosis while inhibits the proliferation of human periodontal ligament stem cells through crosstalk between Erk and Bcl-2 signaling pathways. Int J Med Sci.

[7] Takeuchi S, Kasamatsu A, Yamatoji M, Nakashima D, Endo-Sakamoto Y, Koide N, Takahara T, Shimizu T, Iyoda M, Ogawara K (2017). TEAD4-YAP interaction regulates tumoral growth by controlling cell-cycle arrest at the G1 phase. Biochem Biophys Res Commun.

[8] Cabochette P, Vega-Lopez G, Bitard J, Parain K, Chemouny R, Masson C, Borday C, Hedderich M, Henningfeld KA, Locker M (2015). YAP controls retinal stem cell DNA replication timing and genomic stability. elife.

[9] Shen Z, Stanger BZ (2015). YAP regulates S-phase entry in endothelial cells. PLoS One.

[10] Yang S, Zhang L, Liu M, Chong R, Ding SJ, Chen Y, Dong J (2013). CDK1 phosphorylation of YAP promotes mitotic defects and cell motility and is essential for neoplastic transformation. Cancer Res.

[11] Li YB, Zhao WL, Wang YX, Zhang CX, Jiang JD, Bi CW, Tang S, Chen RX, Shao RG, Song DQ (2013). Discovery, synthesis and biological evaluation of cycloprotoberberine derivatives as potential antitumor agents. Eur J Med Chem.

[12] Ha GH, Kim HS, Lee CG, Park HY, Kim EJ, Shin HJ, Lee JC, Lee KW, Lee CW (2009). Mitotic catastrophe is the predominant response to histone acetyltransferase depletion. Cell Death Differ.

[13] Guo L, Liu X, Nishikawa K, Plunkett W (2007). Inhibition of topoisomerase IIalpha and G2 cell cycle arrest by NK314, a novel benzo[c]phenanthridine currently in clinical trials. Mol Cancer Ther.

[14] Zhao W, He H, Ren K, Zhang H, Chen Y, Shao R (2013). Myofibrillogenesis regulator-1 promotes cell adhesion and migration in human hepatoma cells. Chin Sci Bull.

[15] Zhao W, Zhang C, Bi C, Ye C, Song D, Liu X, Shao R (2016). Sophoridinol derivative 05D induces tumor cells apoptosis by topoisomerase1-mediated DNA breakage. Onco Targets Ther.

[16] Zhao W, Jiang G, Bi C, Li Y, Liu J, Ye C, He H, Li L, Song D, Shao R (2015). The dual topoisomerase inhibitor A35 preferentially and specially targets topoisomerase 2alpha by enhancing pre-strand and post-strand cleavage and inhibiting DNA religation. Oncotarget.

[17] Braselmann H, Michna A, Hess J, Unger K (2015). CFAssay: statistical analysis of the colony formation assay. Radiat Oncol.

[18] Katz D, Ito E, Lau KS, Mocanu JD, Bastianutto C, Schimmer AD, Liu FF (2008). Increased efficiency for performing colony formation assays in 96-well plates: novel applications to combination therapies and high-throughput screening. BioTechniques.

[19] Liu J, Mao W, Ding B, Liang CS (2008). ERKs/p53 signal transduction pathway is involved in doxorubicin-induced apoptosis in H9c2 cells and cardiomyocytes. Am J Physiol Heart Circ Physiol.

[20] Tewey KM, Rowe TC, Yang L, Halligan BD, Liu LF (1984). Adriamycin-induced DNA damage mediated by mammalian DNA topoisomerase II. Science.

[21] Zhou BB, Elledge SJ (2000). The DNA damage response: putting checkpoints in perspective. Nature.

[22] Mantena SK, Sharma SD, Katiyar SK (2006). Berberine, a natural product, induces G1-phase cell cycle arrest and caspase-3-dependent apoptosis in human prostate carcinoma cells. Mol Cancer Ther.

[23] Rogakou EP, Pilch DR, Orr AH, Ivanova VS, Bonner WM (1998). DNA double-stranded breaks induce histone H2AX phosphorylation on serine 139. J Biol Chem.

[24] Sharma A, Singh K, Almasan A (2012). Histone H2AX phosphorylation: a marker for DNA damage. Methods Mol Biol.

[25] Kuo LJ, Yang LX (2008). Gamma-H2AX - a novel biomarker for DNA double-strand breaks. In Vivo.

[26] Ben-David U, Cowell IG, Austin CA, Benvenisty N (2014). Controlling the survival of human pluripotent stem cells by small molecule-based targeting of topoisomerase II alpha. Stem Cells.

[27] Chen T, Sun Y, Ji P, Kopetz S, Zhang W (2014). Topoisomerase IIalpha in chromosome instability and personalized cancer therapy. Oncogene.

[28] Graves PR, Yu L, Schwarz JK, Gales J, Sausville EA, O'Connor PM, Piwnica-Worms H (2000). The Chk1 protein kinase and the Cdc25C regulatory pathways are targets of the anticancer agent UCN-01. J Biol Chem.

[29] Sampath D, Cortes J, Estrov Z, Du M, Shi Z, Andreeff M, Gandhi V, Plunkett W (2006). Pharmacodynamics of cytarabine alone and in combination with 7-hydroxystaurosporine (UCN-01) in AML blasts in vitro and during a clinical trial. Blood.

[30] Hotte SJ, Oza A, Winquist EW, Moore M, Chen EX, Brown S, Pond GR, Dancey JE, Hirte HW (2006). Phase I trial of UCN-01 in combination with topotecan in patients with advanced solid cancers: a Princess Margaret hospital phase II consortium study. Ann Oncol.

[31] Moon S, Kim W, Kim S, Kim Y, Song Y, Bilousov O, Kim J, Lee T, Cha B, Kim M (2017). Phosphorylation by NLK inhibits YAP-14-3-3-interactions and induces its nuclear localization. EMBO Rep.

[32] Keshet R, Adler J, Ricardo Lax I, Shanzer M, Porat Z, Reuven N, Shaul Y (2015). C-Abl antagonizes the YAP oncogenic function. Cell Death Differ.

[33] Cottini F, Hideshima T, Xu C, Sattler M, Dori M, Agnelli L, ten Hacken E, Bertilaccio MT, Antonini E, Neri A (2014). Rescue of Hippo coactivator YAP1 triggers DNA damage-induced apoptosis in hematological cancers. Nat Med.

[34] Zhao W, He H, Ren K, Li B, Zhang H, Lin Y, Shao RG (2013). MR-1 blocks the megakaryocytic differentiation and transition of CML from chronic phase to blast crisis through MEK dephosphorylation. Blood Cancer J.

[35] Harvey KF, Zhang X, Thomas DM (2013). The hippo pathway and human cancer. Nat Rev Cancer.

[36] Ravizza R, Gariboldi MB, Passarelli L, Monti E (2004). Role of the p53/p21 system in the response of human colon carcinoma cells to doxorubicin. BMC Cancer.

[37] Taylor WR, Stark GR (2001). Regulation of the G2/M transition by p53. Oncogene.

[38] Williams AB, Schumacher B. p53 in the DNA-Damage-Repair Process. Cold Spring Harb Perspect Med. 2016;6(5). 10.1101/cshperspect.a026070. 10.1101/cshperspect.a026070 PMC4852800 27048304

[39] Gerić M, Štraser A, Gajski G, Nunić J, Žegura B, Filipič M, Garaj-Vrhovac V (2013). Use of γ-H2AX foci assay on human peripheral blood lymphocytes as sensitive biomarker of exposure. 9th symposium of the Croatian radiation protection association: 2013.

[40] Lakin ND, Jackson SP (1999). Regulation of p53 in response to DNA damage. Oncogene.

[41] Pan D (2010). The hippo signaling pathway in development and cancer. Dev Cell.

